# Perspectives of Anatomy Education Among Osteopathic Medical Students: A Cross-Sectional Survey

**DOI:** 10.7759/cureus.105031

**Published:** 2026-03-11

**Authors:** Robert J Heins, Matthew Mindrup, Justine Hemaya, Sara S Sloan

**Affiliations:** 1 Department of Anatomical Sciences, Kansas City University College of Osteopathic Medicine, Kansas City, USA

**Keywords:** anatomy education, medical education research, medical licensure examination, osteopathic medical education, preclinical medical education

## Abstract

Background

Anatomy education is a foundational component of osteopathic medical training and contributes to clinical development. While prior studies have examined objective outcomes of anatomy curricular change, osteopathic medical students’ perceptions of anatomy education, including perceived retention, licensure preparedness, and influence on career interests, remain incompletely characterized.

Methods

A 30-question electronic survey was distributed to second-, third-, and fourth-year osteopathic medical students in the United States between October 2024 and March 2025. Survey domains included didactic and laboratory anatomy instruction, perceived retention of anatomical knowledge, perceived preparedness for the Comprehensive Osteopathic Medical Licensing Examination (COMLEX) Level 1 and the United States Medical Licensing Examination (USMLE) Step 1, and perceived influence on specialty interests. Descriptive statistics and nonparametric tests were used for analysis. Descriptive statistics were calculated for all survey items. Nonparametric tests were used for group comparisons. The Kruskal-Wallis test was applied for comparisons across three groups, and the Mann-Whitney U test for two-group comparisons. Statistical significance was defined as p < 0.05.

Results

Of 125 respondents, 116 met the inclusion criteria. Most respondents perceived their anatomy education as valuable and relevant to their training. Many reported moderate or greater perceived retention of anatomical knowledge more than one year after instruction, with no significant differences across class years. Students who perceived greater value in their anatomy education were more likely to report feeling prepared for licensure examinations. Perceived preparedness was higher for COMLEX Level 1 than for USMLE Step 1. Students pursuing surgical specialties reported a greater perceived influence of anatomy education on career interests compared with those pursuing nonsurgical fields.

Conclusion

Osteopathic medical students commonly perceive anatomy education as valuable and relevant, though perceptions of retention, licensure preparedness, and career influence vary. These findings reflect learner perceptions rather than objective outcomes and may inform future hypothesis-generating research and curricular considerations in osteopathic medical education. Considerations may include the integration of clinically oriented instruction, longitudinal reinforcement of anatomical concepts, and innovative teaching strategies to enhance learner engagement and perceived relevance.

## Introduction

Anatomy education is a fundamental component of osteopathic medical training, providing a foundation for clinical practice. A thorough understanding of human anatomy underpins safe and effective medical and surgical care, informing physical examination, diagnostic reasoning, and procedural decision-making across clinical disciplines. Since 2010, anatomy education has undergone significant changes in part because of the Carnegie Foundation’s call for curriculum reform in medical schools [1,2]. As a result, medical students have been exposed to varying instructional methods due to evolving educational research and curriculum delivery models. One notable trend has been a reduction in anatomy laboratory hours devoted to cadaveric dissection [3,4], with increased reliance on virtual dissection platforms and online learning resources. These shifts were further accelerated by curricular adaptations during the COVID-19 pandemic [4-6]. Recent studies conducted during and after the COVID-19 pandemic have documented substantial transitions toward distance and hybrid anatomy education, with students reporting mixed perceptions of educational value and engagement, particularly regarding reduced hands-on laboratory exposure [7]. A 2022 analysis at Rowan University School of Osteopathic Medicine similarly found that students participating in hands-on dissection reported more favorable perceptions of their anatomy education compared with those learning through virtual modalities [8].

Anatomical knowledge serves as a basis for clinical disciplines, such as surgery, radiology, physical examination, and diagnostic reasoning, throughout a physician’s career [9]. Despite its importance, prior studies have reported dissatisfaction among medical students regarding anatomy instruction [10-13]. More recent student-centered surveys continue to demonstrate variability in satisfaction with anatomy education, particularly in relation to instructional modality, laboratory access, and perceived preparedness for clinical application, suggesting that student perceptions remain heterogeneous in contemporary curricula [14,15].

Some reported inadequacies in mastering anatomy may relate to the temporal gap between preclinical anatomy instruction and later clinical application or licensure examinations. Jurjus et al. demonstrated declining anatomical knowledge retention between the first and third years of medical school [16]. This has raised questions regarding the need for longitudinal reinforcement of anatomy education. Contemporary studies have similarly highlighted concerns about long-term retention of anatomical knowledge, particularly in curricula with condensed preclinical anatomy instruction, though much of this work has focused on objective assessments rather than learner-reported perceptions [14,15]. Dee et al. reported that supplemental surgical anatomy instruction in later training years was associated with increased student interest and perceived retention [17].

Anatomy content comprises approximately 11-15% of the United States Medical Licensing Examination (USMLE) Step 1 examination [18] and over 15% of the Comprehensive Osteopathic Medical Licensing Examination (COMLEX) Level 1 [19]. Prior research has examined associations between anatomy curricula and licensure examination performance [20,21]. However, limited recent literature has focused specifically on osteopathic medical students’ perceptions of their preparedness for both COMLEX and USMLE examinations. Despite ongoing curricular reform and expanded use of blended instructional approaches, few contemporary studies have examined osteopathic medical students’ perceptions of anatomy education, including perceived retention, licensure preparedness, and influence on career interests. Understanding these perceptions may help inform curricular design and guide educational strategies aimed at improving anatomy training in osteopathic medical education.

This study aimed to assess osteopathic medical students’ perceptions of anatomy education, their perceived preparedness for licensure examinations, and the perceived influence of anatomy instruction on career interests.

## Materials and methods

This study employed a cross-sectional, anonymous survey design to assess osteopathic medical students’ perceptions of anatomy education. Institutional Review Board approval was obtained from Kansas City University (IRB #2227275-1). Participation was voluntary, and informed consent was obtained electronically prior to survey initiation through an introductory consent page. Responses were collected anonymously, and no identifying information was recorded. No incentives were offered for participation.

A 30-question survey was developed de novo to evaluate students’ experiences and perceptions related to anatomy education during medical school. Survey domains were informed by a focused review of the anatomy education literature and were designed to capture perceptions of curricular structure, didactic and laboratory instructional modalities, perceived retention of anatomical knowledge, preparedness for medical licensure examinations, and perceived influence of anatomy education on specialty interest. The survey was developed collaboratively by all study authors, including three osteopathic medical students and one faculty member in anatomy at an osteopathic medical school. Items were intentionally perception-based and did not assess objective educational outcomes. Survey development and reporting were informed by established guidelines for internet-based surveys, including the Checklist for Reporting Results of Internet E-Surveys (CHERRIES) checklist [22].

Most survey items used a five-point Likert scale, with response anchors adapted to the construct being measured (e.g., strongly disagree to strongly agree; very inadequate to very adequate; never to always). Higher Likert values consistently reflected greater agreement, adequacy, or frequency. Pilot testing was conducted with the research team and colleagues at an osteopathic medical school to assess clarity, readability, and content relevance. No formal psychometric validation or reliability testing was performed. The complete survey instrument is provided in the Appendix.

Eligible participants included second-, third-, and fourth-year osteopathic medical students enrolled at U.S. colleges of osteopathic medicine during the study period. First-year students and students enrolled at allopathic medical schools were excluded. The survey was distributed electronically between October 2024 and March 2025 using Qualtrics XM (Qualtrics, LLC, Provo, Utah, US) via school-specific email lists and national osteopathic student organization email lists. Institutional affiliation of respondents was not collected to preserve maximum anonymity; therefore, the number of represented colleges of osteopathic medicine could not be determined.

**Figure 1:**
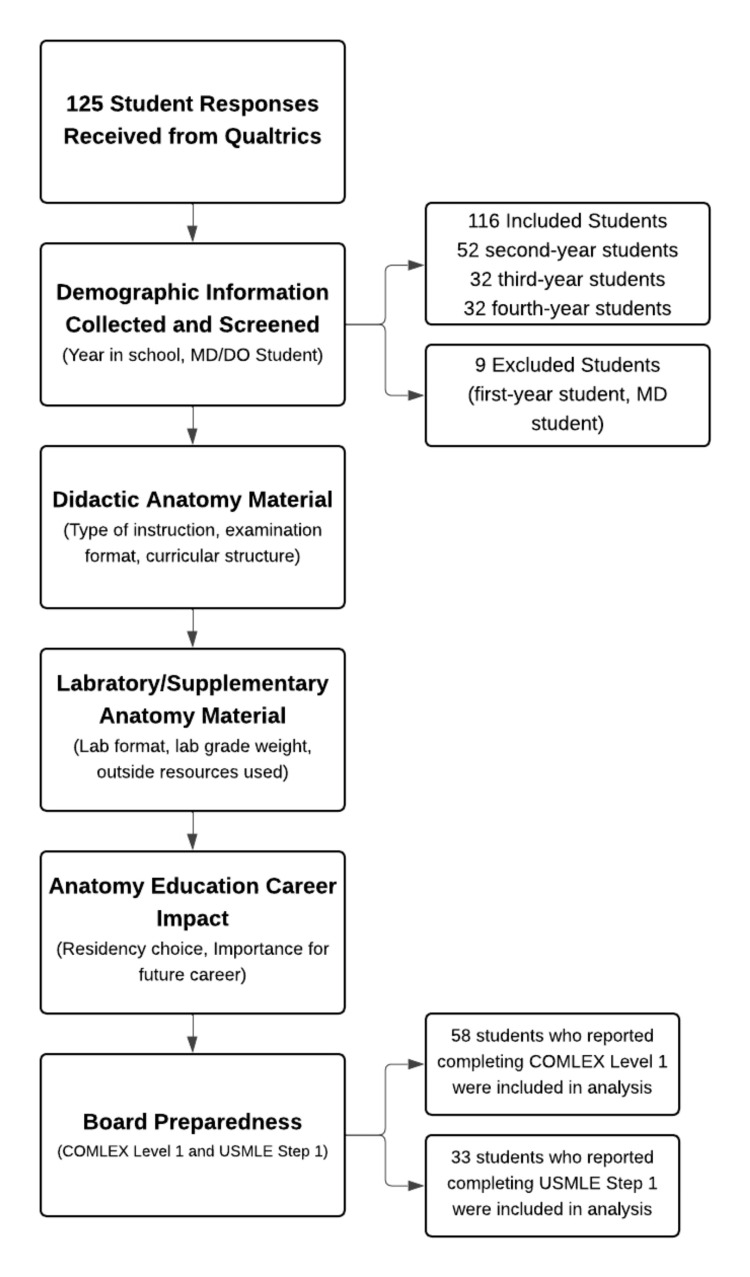
Participant screening and progression through major survey domains

A total of 125 responses were received (Figure 1). After exclusion of responses from first-year students and students enrolled at allopathic medical schools, 116 responses were included in the final analysis. The final sample included second-, third-, and fourth-year osteopathic medical students. Due to item-level nonresponse, the number of respondents varied across survey questions, particularly for items related to licensure examinations that were only applicable to students who had completed COMLEX Level 1 or USMLE Step 1.

Survey data were analyzed using Qualtrics XM and SPSS software (version 29.0.2.0; IBM Corp., Armonk, NY, US). Descriptive statistics were calculated for demographic variables and survey responses, including frequencies and percentages for categorical data. Likert-scale responses were analyzed using nonparametric statistical tests. The Mann-Whitney U test was used for comparisons between two independent groups, and the Kruskal-Wallis test was used for comparisons across three or more groups. For selected analyses, response categories were collapsed to facilitate interpretation. A two-sided significance level of p < 0.05 was used for all analyses.

## Results

Didactic anatomy material

A total of 86.1% of respondents reported that anatomy instruction at their institution was delivered within a systems-based curriculum rather than as a standalone course. Students most commonly indicated that primary anatomy instruction relied on lecture-based teaching or a combination of lecture and problem-based learning, with assessment typically consisting of multiple-choice examinations, laboratory practicals, or both. Additionally, 62.9% of respondents indicated that they supplemented their anatomy education with resources outside of those provided by their institution. Detailed distributions of instructional structure, instructional modalities, assessment formats, and reported use of supplemental resources are presented in Tables 1-2.

**Table 1:** Reported instructional methods, assessment formats, and laboratory practices

Survey Item	Response Option	Frequency (%)	n
Primary anatomy integration	Integrated (systems-based)	86.1%	93
	Standalone anatomy course	13.9%	15
Primary anatomy teaching methods	Lecture	49.1%	52
	Lecture + problem-based learning (PBL)	46.2%	49
	PBL	4.7%	5
Method of assessment	Multiple choice + lab practical	69.1%	74
	Multiple choice only	15.9%	17
	Lab practical only	15.0%	16
Anatomy laboratory modality	Cadaveric dissection	92.3%	96
	Cadaveric prosection	3.8%	4
	No lab/prefer not to say	2.9%	3
	Virtual	1.0%	1
Laboratory attendance policy	Required	95.0%	96
	Optional	5.0%	5
Percentage of course grade from lab practical	25–50%	48.5%	48
	10–25%	31.3%	31
	50–75%	10.1%	10
	<10%	8.1%	8
	75–100%	2.0%	2

**Table 2:** Selected survey items related to students’ subjective perceptions of anatomy education ^a^Response options were based on a 5-point Likert scale, though wording varied by item (e.g., “Strongly disagree” to “Strongly agree,” “Never” to “Always,” or “Very inadequate” to “Very adequate”). Higher values consistently indicate greater agreement or frequency. Only questions addressing self-reported experiences, attitudes, or beliefs were included. Not all items from the full 30-question survey are shown. The complete survey instrument and the full distribution of responses for each item are provided in the Appendix. COMLEX: Comprehensive Osteopathic Medical Licensing Examination; USMLE: United States Medical Licensing Examination

Survey Question	Responses ^a^					Total number of responses	Median response
	1 (Low)	2	3	4	5 (High)		
The teaching method employed in the anatomy laboratory at my institution proved to be the most effective option for me	13	10	9	33	36	101	4
I have retained the anatomical material taught in my medical school courses	3	24	9	51	18	105	4
How often did you find yourself needing to supplement your anatomy learning with outside resources due to incomplete/ineﬀective teaching from your university	2	37	36	22	8	105	3
I was/will be adequately prepared for surgical rotations from the anatomical education I received in medical school	2	18	15	45	23	103	4
My anatomical education in medical school was a valuable experience	0	5	7	24	68	104	5
I would have benefitted by having additional resources provided by my school to supplement my anatomy board preparation (gross anatomy review, high-yield anatomy board review sessions, third-party resources)	5	13	28	38	19	103	4
Continuing to learn anatomy will be important in my career as a physician	1	2	9	28	63	103	4
The teaching method employed in the anatomy laboratory at my institution proved to be the most eﬀective option for me	13	10	9	33	36	101	5
How much did the anatomy education oﬀered at your institution influence the medical specialty or specialties that you are interested in pursuing	37	16	28	18	4	103	4
Rank how prepared you felt the anatomical education provided by your institution was for COMLEX Level 1 (Must have already taken Level 1 at the time of survey)	0	7	9	29	13	58	4
Rank how adequate the anatomical education provided by your institution was for passing COMLEX Level 1 on your first attempt (Must have already taken Level 1 at the time of survey)	0	5	8	31	14	58	4
Rank how prepared you felt the anatomical education provided by your institution was for USMLE Step 1 (Must have already taken Step 1 at the time of survey)	1	4	5	15	8	33	4
Rank how adequate the anatomical education provided by your institution was for passing USMLE Step 1 on your first attempt (Must have already taken Step 1 at the time of survey	0	3	4	18	8	33	4

Laboratory anatomy material

A total of 92.3% respondents reported participation in cadaveric dissection as the primary anatomy laboratory modality, with smaller proportions reporting cadaveric prosection, virtual laboratory instruction, or no laboratory component. Laboratory attendance was required in 95% of surveyed students, and laboratory practical examinations contributed a substantial portion of the overall anatomy course grade. Additional details regarding laboratory instructional modalities, attendance policies, and grading structures are summarized in Table 1.

A total of 68.1% respondents indicated that they perceived the instructional methods used in their anatomy laboratory as the most effective learning modality for them. Frequencies of student responses related to perceived laboratory effectiveness and use of supplemental learning resources are reported in Table 2.

Anatomy education and career preparation

A total of 88.5% of students indicated that they perceived their anatomy education as a valuable component of medical school training and reported that continued learning of anatomy would remain important in their future careers as physicians. Many students (66%) also reported a moderate to strong perceived preparedness for third- and fourth-year surgical rotations based on their anatomy education.

**Figure 2:**
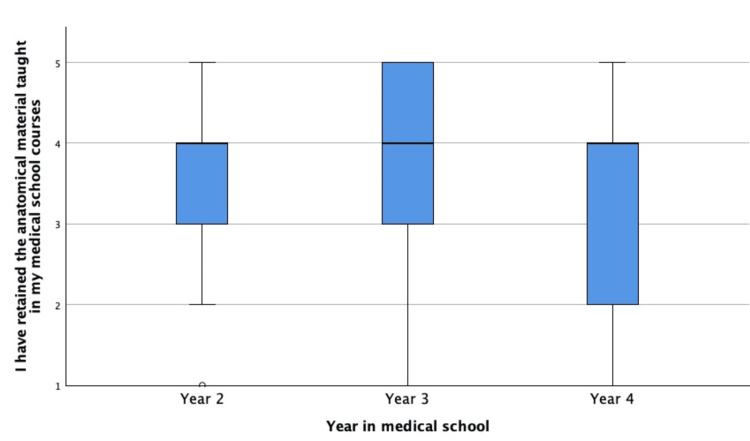
Distribution of reported anatomy education retention by class year in medical school 1: strongly disagree, 2: disagree, 3: neither agree nor disagree, 4: somewhat agree 5: agree In the box plots, the central line represents the median response, the box indicates the interquartile range (25th-75th percentiles), and the whiskers represent the range of responses.

With respect to retention, 65.7% of respondents reported a moderate or greater perceived retention of anatomical material learned during prior medical school coursework. When perceptions of retention were stratified by class year, no statistically significant differences were observed between second-, third-, and fourth-year students. The distribution of self-reported retention across class years is illustrated in Figure 2.

Confidence in licensure examination preparedness

Among students who had completed COMLEX Level 1 or USMLE Step 1, 77.5% reported feeling adequately or very adequately prepared for COMLEX Level 1, while 69.7% reported similar levels of preparedness for USMLE Step 1 based on their anatomy education. Students who rated their anatomy education as more valuable were significantly more likely to report greater perceived preparedness for both examinations. There were no statistically significant differences in perceived preparedness between students who reported frequent use of outside resources and those who did not. Response distributions for licensure-related survey items are presented in Table 2.

Impact of anatomy education on specialty choice

When asked about specialty interests, 55.8% of respondents reported nonsurgical career intentions, 31.7% reported interest in surgical specialties, and 12.5% were undecided at the time of the survey. Nearly half of respondents (49.0%) indicated that anatomy education had at least a moderate self-reported influence on their specialty interest. Among students pursuing surgical specialties, 66.7% reported that their anatomy laboratory experience had at least a moderate influence on their career choice, compared with 48.4% of those pursuing nonsurgical fields, and this difference was statistically significant (p = 0.021). Distributions of responses related to specialty interest and perceived influence are summarized in Table 2.

## Discussion

This study examined osteopathic medical students’ perceptions of anatomy education, focusing on perceived educational value, retention of anatomical knowledge, preparedness for licensure examinations, and influence on career interests. Overall, respondents commonly reported that anatomy education was a valuable component of their medical training and that continued engagement with anatomy would remain important in their future careers as physicians. These findings are consistent with prior work emphasizing the perceived foundational role of anatomy in medical education and align with more recent studies demonstrating sustained student valuation of anatomy despite evolving instructional formats [12,14].

Students reported varying perceptions regarding retention of anatomical knowledge following the completion of preclinical coursework. While many respondents indicated moderate or greater perceived retention, no statistically significant differences were observed across class years. This suggests that perceived retention may remain relatively stable across training stages, although the perception-based nature of the data limits conclusions regarding actual knowledge retention. Recent anatomy education research has suggested that learner perceptions of retention may diverge from objective performance measures, particularly in curricula emphasizing early, condensed anatomy instruction, underscoring the importance of distinguishing perceived confidence from demonstrable knowledge [14,15]. Although respondents in this study reported relatively stable perceived retention of anatomical knowledge across training years, prior studies have demonstrated objective declines in anatomical knowledge over time [13]. One potential strategy to support long-term retention may be greater longitudinal reinforcement of anatomy throughout clinical training, particularly through integration with clinical pathology, imaging, and procedural learning rather than reliance on early preclinical memorization alone.

With respect to licensure examinations, students who reported greater perceived value in their anatomy education were more likely to report feeling prepared for COMLEX Level 1 and USMLE Step 1. Importantly, these findings reflect self-reported confidence rather than objective examination performance or curricular effectiveness. Differences in perceived preparedness between examinations should therefore be interpreted cautiously and understood as subjective perceptions rather than indicators of differential curricular alignment. Although prior studies have examined objective relationships between anatomy curricula and licensure examination performance [20,21], such outcomes were not assessed in the present study.

Anatomy education was also perceived to influence specialty interests for some respondents, particularly among students pursuing surgical fields. Students interested in surgical specialties reported a greater self-reported influence of anatomy education on career considerations compared with those pursuing nonsurgical paths. These findings align with prior literature suggesting that exposure to anatomy education, particularly laboratory-based experiences, may shape students’ perceptions of specialty relevance [17,23,24]. Recent student-focused studies similarly report that anatomy instructional modality and access to laboratory experiences may influence how learners perceive the relevance of anatomy to future clinical roles, including surgical careers, while emphasizing perception rather than causation [14].

These results suggest that osteopathic medical students commonly perceive anatomy education as meaningful and relevant to both clinical training and career development. The findings highlight areas where students perceive benefits, as well as areas where confidence and perceived retention may vary, underscoring the importance of understanding learner perspectives when considering curricular structure and reinforcement strategies.

Limitations

Several limitations warrant consideration when interpreting the results. This study relied on self-reported perceptions, which may be influenced by recall bias, response bias, or individual interpretation of survey items. Additionally, the findings reflect subjective perceptions rather than objective measures of anatomical knowledge retention, curricular effectiveness, or licensure examination performance. Participation was voluntary, and survey distribution was electronic, introducing potential self-selection bias. Students who chose to participate may have differed in their perceptions of anatomy education compared with those who did not respond. The number of respondents represents a small proportion of the national osteopathic medical student population, which limits generalizability. Institutional affiliation was not collected to preserve respondent anonymity, preventing the assessment of institutional representation and potential clustering of responses. The survey instrument was developed de novo and, although pilot tested for clarity and relevance, was not formally validated. Subgroup analyses, including comparisons across class years and specialty interests, may have been underpowered to detect small differences and should be interpreted cautiously.

## Conclusions

In this survey, osteopathic medical students commonly perceived anatomy education as a valuable and relevant component of their training, with continued importance for future clinical practice. Students reported variable perceptions regarding retention of anatomical knowledge, preparedness for licensure examinations, and the influence of anatomy education on career interests. These findings reflect student perceptions rather than objective educational outcomes. Understanding learner perspectives may help inform future hypothesis-generating research and guide consideration of curricular reinforcement strategies within osteopathic medical education, including the integration of clinically oriented instruction, longitudinal reinforcement of anatomical concepts, and the use of innovative teaching strategies to enhance learner engagement and perceived relevance.

## Appendices

**Table 3:** Distribution of responses for the full anatomy education survey Values are presented as a number (percentage). Percentages for select-all-that-apply questions may exceed 100%.

Question	Response	n (%)
Please indicate whether you attend an Allopathic (MD) or Osteopathic (DO) medical school located in the United States	Allopathic (MD)	1 (0.81)
	Osteopathic (DO)	122 (99.19)
	I do not attend a medical school located in the United States	0 (0.00)
	Prefer not to say	0 (0.00)
Year in medical school	Year 1	6 (4.88)
	Year 2	53 (43.09)
	Year 3	32 (26.02)
	Year 4	32 (26.02)
	Prefer not to say	0 (0.00)
How is primary anatomy material incorporated into pre-clinical/didactic courses at your institution	Integrated into other courses (systems-based)	94 (86.24)
	Freestanding anatomy course	15 (13.76)
What year(s) in school is primary anatomy material taught (select all that apply)	Year 1	108 (77.70)
	Year 2	31 (22.30)
	Year 3	0 (0.00)
	Year 4	0 (0.00)
How many weeks is the primary anatomy course at your institution	<5 weeks	1 (0.95)
	5–10 weeks	2 (1.90)
	10–15 weeks	4 (3.81)
	15–20 weeks	12 (11.43)
	>20 weeks	86 (81.90)
What methods of instruction are used to teach primary anatomy material at your institution	Lecture	52 (48.60)
	Problem-based learning	5 (4.67)
	Both lecture & problem-based learning	50 (46.73)
What is the attendance policy for primary anatomy material at your institution	Required	96 (88.89)
	Optional	12 (11.11)
What method of assessment is used for primary anatomy material at your institution	Multiple-choice examination	17 (15.74)
	Lab practical	16 (14.81)
	Multiple-choice examination & lab practical	75 (69.44)
What methods of instruction are used to teach additional anatomy material at your institution (select all that apply)	Physiology courses	88 (24.31)
	Pathology courses	81 (22.38)
	Physical exam & clinical skills	93 (25.69)
	Osteopathic principles & practices (OMM/OMT)	99 (27.35)
	My institution does not provide additional instruction in anatomy	1 (0.28)
What is the main modality of instruction for the anatomy laboratory at your institution	Cadaveric dissection	97 (92.38)
	Cadaveric Prosection	4 (3.81)
	Virtual	1 (0.95)
	My institution does not have an anatomy laboratory	2 (1.90)
	Prefer not to say	1 (0.95)
What percentage of the overall course grade are anatomy lab practical score(s) at your institution	<10%	8 (8.00)
	10–25%	31 (31.00)
	25–50%	49 (49.00)
	50–75%	10 (10.00)
	75–100%	2 (2.00)
The teaching method employed in the anatomy laboratory at my institution proved to be the most effective option for me	Strongly disagree	13 (19.70)
	Somewhat disagree	10 (15.15)
	Neither agree nor disagree	10 (15.15)
	Somewhat agree	33 (50.00)
On average, what percentage of your weekly studying is/was devoted to anatomy-related material	<10%	7 (6.60)
	10–25%	42 (39.62)
	25–50%	41 (38.68)
	50–75%	13 (12.26)
	75–100%	3 (2.83)
I have retained the anatomical material taught in my medical school courses	Strongly disagree	3 (2.83)
	Somewhat disagree	24 (22.64)
	Neither agree nor disagree	9 (8.49)
	Somewhat agree	52 (49.06)
	Strongly agree	18 (16.98)
How often did you find yourself needing to supplement your anatomy learning with outside resources due to incomplete/ineffective teaching from your university	Never	2 (1.89)
	Rarely	37 (34.91)
	Sometimes	36 (33.96)
	Often	22 (20.75)
	Always	9 (8.49)
I was/will be adequately prepared for surgical rotations from the anatomical education I received in medical school	Strongly disagree	2 (1.92)
	Somewhat disagree	18 (17.31)
	Neither agree nor disagree	16 (15.38)
	Somewhat agree	45 (43.27)
	Strongly agree	23 (22.12)
My anatomical education in medical school was a valuable experience	Strongly disagree	0 (0.00)
	Somewhat disagree	5 (4.76)
	Neither agree nor disagree	7 (6.67)
	Somewhat agree	25 (23.81)
	Strongly agree	68 (64.76)
I would have benefited by having additional resources provided by my school to supplement my anatomy board preparation	Strongly disagree	5 (4.81)
	Somewhat disagree	13 (12.50)
	Neither agree nor disagree	28 (26.92)
	Somewhat agree	38 (36.54)
	Strongly agree	20 (19.23)
Which additional resources would have benefited your anatomy board preparation (select all that apply)	Cadaveric gross anatomy review	34 (14.66)
	More anatomy integrated into the curriculum	26 (11.21)
	High-yield anatomy board review sessions	73 (31.47)
	Third-party resources	47 (20.26)
	Anatomy research opportunities	33 (14.22)
	Anatomy fellowship/internship/tutoring	19 (8.19)
Continuing to learn anatomy will be important in my career as a physician	Strongly disagree	1 (0.96)
	Somewhat disagree	3 (2.88)
	Neither agree nor disagree	9 (8.65)
	Somewhat agree	28 (26.92)
	Strongly agree	63 (60.58)
What medical specialty/specialties are you interested in pursuing	Non‑surgical medical specialty	58 (55.77)
	Surgical	33 (31.73)
	Unsure	13 (12.50)
How much did the anatomy education offered at your institution influence the specialty you are interested in pursuing	No influence	37 (35.58)
	Low influence	16 (15.38)
	Moderate influence	29 (27.88)
	Strong influence	18 (17.31)
	High influence	4 (3.85)
Did you take the COMLEX Level 1 examination	Yes	59 (56.73)
	No	44 (42.31)
	Prefer not to say	1 (0.96)
Rank how prepared you felt the anatomical education provided by your institution was for COMLEX Level 1	Not prepared	0 (0.00)
	Somewhat prepared	7 (12.07)
	Moderately prepared	9 (15.52)
	Prepared	29 (50.00)
	Very prepared	13 (22.41)
Rank how adequate the anatomical education provided by your institution was for passing COMLEX Level 1 on the first attempt	Did not pass the first attempt	0 (0.00)
	Inadequate	0 (0.00)
	Somewhat inadequate	5 (8.62)
	Neutral	8 (13.79)
	Adequate	31 (53.45)
	Very adequate	14 (24.14)
Did you take the USMLE Step 1 Examination	Yes	33 (31.73)
	No	69 (66.35)
	Prefer not to say	2 (1.92)
Rank how prepared you felt the anatomical education provided by your institution was for USMLE Step 1	Not prepared	1 (3.03)
	Somewhat prepared	4 (12.12)
	Moderately prepared	5 (15.15)
	Prepared	15 (45.45)
	Very prepared	8 (24.24)
Rank how adequate the anatomical education provided by your institution was for passing USMLE Step 1 on the first attempt	Did not pass the first attempt	0 (0.00)
	Inadequate	0 (0.00)
	Somewhat inadequate	3 (9.09)
	Neutral	4 (12.12)
	Adequate	18 (54.55)
	Very adequate	8 (24.24)

## References

[1] Irby DM, Cooke M, O’Brien BC (2010). Calls for reform of medical education by the Carnegie Foundation for the Advancement of Teaching: 1910 and 2010. Acad Med.

[2] Hegazy AA (2024). Human anatomy is the geography of medical practice: scope and teaching. Med J Dr. D Y Patil Vidyapeeth.

[3] McBride JM, Drake RL (2018). National survey on anatomical sciences in medical education. Anat Sci Educ.

[4] Shin M, Prasad A, Sabo G, Macnow AS, Sheth NP, Cross MB, Premkumar A (2022). Anatomy education in US medical schools: before, during, and beyond COVID-19. BMC Med Educ.

[5] Iwanaga J, Loukas M, Dumont AS, Tubbs RS (2021). A review of anatomy education during and after the COVID-19 pandemic: revisiting traditional and modern methods to achieve future innovation. Clin Anat.

[6] Franchi T (2020). The impact of the COVID‐19 pandemic on current anatomy education and future careers: a student’s perspective. Anat Sci Educ.

[7] Özen KE, Erdoğan K, Malas MA (2022). Evaluation of views and perceptions of the medical faculty students about distance anatomy education during the COVID-19 pandemic. Surg Radiol Anat.

[8] Kochhar S, Tasnim T, Gupta A (2023). Is cadaveric dissection essential in medical education? A qualitative survey comparing pre-and post-COVID-19 anatomy courses. J Osteopath Med.

[9] Saverino D (2021). Teaching anatomy at the time of COVID-19. Clin Anat.

[10] Sabesan VJ, Schrotenboer A, Habeck J, Lombardo D, Stine S, Jildeh TR, Meiyappan A (2018). Musculoskeletal education in medical schools: a survey of allopathic and osteopathic medical students. J Am Acad Orthop Surg Glob Res Rev.

[11] Bhangu A, Boutefnouchet T, Yong X, Abrahams P, Joplin R (2010). A three-year prospective longitudinal cohort study of medical students' attitudes toward anatomy teaching and their career aspirations. Anat Sci Educ.

[12] Fitzgerald JE, White MJ, Tang SW, Maxwell-Armstrong CA, James DK (2008). Are we teaching sufficient anatomy at medical school? The opinions of newly qualified doctors. Clin Anat.

[13] Triepels CP, Koppes DM, Van Kuijk SM (2018). Medical students' perspective on training in anatomy. Ann Anat.

[14] Aydrose A, Elsdaig H, Abdalla A, Abdelsadig MK, Elderderi D (2025). Medical students’ perception toward various human anatomy teaching methods in Khartoum, Sudan. Cureus.

[15] Barut C, Ogut E, Karaer E, Yavuz M (2025). Anatomy study preferences of medical students in relation to gender, academic year and geographical distribution: considerations for anatomy education approaches. Bratisl Med J.

[16] Jurjus RA, Lee J, Ahle S, Brown KM, Butera G, Goldman EF, Krapf JM (2014). Anatomical knowledge retention in third-year medical students prior to obstetrics and gynecology and surgery rotations. Anat Sci Educ.

[17] Dee EC, Alty IG, Agolia JP (2021). A surgical view of anatomy: perspectives from students and instructors. Anat Sci Educ.

[18] (2024). Step 1 content outline and specifications USMLE. https://www.usmle.org/prepare-your-exam/step-1-materials/step-1-content-outline-and-specifications.

[19] (2024). Blueprint. NBOME. https://www.nbome.org/assessments/comlex-usa/comlex-usa-level-1/blueprint/.

[20] Cuddy MM, Swanson DB, Drake RL, Pawlina W (2013). Changes in anatomy instruction and USMLE performance: empirical evidence on the absence of a relationship. Anat Sci Educ.

[21] Vasan NS, Holland BK (2003). Increased clinical correlation in anatomy teaching enhances students’ performance in the course and National Board subject examination. Med Sci Monit.

[22] Eysenbach G (2004). Improving the quality of web surveys: the Checklist for Reporting Results of Internet E-Surveys (CHERRIES). J Med Internet Res.

[23] Bogomolova K, Hierck BP, van der Hage JA, Hovius SE (2020). Anatomy dissection course improves the initially lower levels of visual-spatial abilities of medical undergraduates. Anat Sci Educ.

[24] Granger NA (2004). Dissection laboratory is vital to medical gross anatomy education. Anat Rec B New Anat.

